# Psychological Distress and Access to Mental Health Services Among Undergraduate Students During the COVID-19 Lockdown in Uganda

**DOI:** 10.3389/fpsyt.2022.792217

**Published:** 2022-06-02

**Authors:** Brandy Nantaayi, Rodney Kato Ndawula, Phillip Musoke, Nelson Ssewante, Lourita Nakyagaba, Joyce Nakiganda Wamala, Emmanuel Arthur Makai, Babrah Wannyana, Nicholas Kisaakye Wamala, Andrew Marvin Kanyike, Gabriel Madut Akech, Daniel Ojilong, Drake Agira, Ann Barbra Nakimuli, Asaph Asiimwe, Felix Bongomin

**Affiliations:** ^1^School of Medicine, College of Health Sciences, Makerere University, Kampala, Uganda; ^2^School of Clinical Medicine and Dentistry, Kampala International University Western, Bushenyi, Uganda; ^3^Faculty of Health Sciences, Busitema University, Mbale, Uganda; ^4^Faculty of Medicine, Gulu University, Gulu, Uganda; ^5^School of Medicine, Uganda Christian University, Kampala, Uganda; ^6^School of Medicine, Soroti University, Soroti, Uganda; ^7^Department of Medical Microbiology and Immunology, Faculty of Medicine, Gulu University, Gulu, Uganda

**Keywords:** psychological distress, depression, access mental health services, COVID-19, lockdown

## Abstract

**Background:**

Lockdown is an important public health approach aimed at curbing the raging effect of the coronavirus disease-2019 (COVID-19). This study aimed at determining the impact of prolonged lockdown on mental health and access to mental health services among undergraduate students in Uganda.

**Methods:**

An online cross-sectional study was conducted anonymously among undergraduates across 10 universities in Uganda. The Distress Questionnaire-5 (DQ-5) and the Patient Health Questionnaire-2 (PHQ-2) were used. Logistic regression analysis was conducted to determine factors associated with psychological distress.

**Results:**

We enrolled 366 participants with a mean age of 24.5 ± 4.6 years. The prevalence of psychological distress was 40.2% (*n* = 147) (cut off 14/25 based on DQ-5) while depression stood at 25.7% (*n* = 94; cut off 3/6 based on PHQ-2) with mean scores of 12.1 ± 4.6 and 1.7 ± 1.6 respectively. Female gender (aOR: 1.6, 95%CI: 1.0–2.6, *p* = 0.032), pursuing a non-medical program (aOR: 2.2, 95%CI: 1.3–3.7, *p* = 0.005) were factors associated with psychological distress while non-medical program (aOR: 2.2, 95%CI: 1.3–3.7, *p* = 0.005) was associated with increased depression. Access to mental health services was associated with both reduced distress (aOR: 0.5, 95%CI: 0.3–0.8, *p* = 0.005) and depression (aOR: 0.6, 95%CI: 0.3–0.9, *p* = 0.034). A majority (65.3%) of the participants reported knowing how to access mental health care and 188 (51.4%) reported having needed emotional support but, only 67 (18.3%) ever sought care from a mental health professional. Of those who had access, only 10 (7%), and 13 (9%) accessed a counselor or a mental health unit, respectively. The barriers to accessibility of mental health care included financial limitations (49.5%), lack of awareness (32.5%), lack of mental health professionals (28.4%), and stigma (13.9%).

**Conclusion:**

Among university students in Uganda during the COVID- 19 lockdown, the burden of psychological distress and depression was substantial. However, access to mental health services was limited by several factors.

## Introduction

As of 9th October 2021, the coronavirus disease-2019 (COVID-19) has affected over 237 million people with over 4.8 million deaths worldwide. Several preventive measures as recommended by the World Health Organization (WHO) have been implemented at personal, family, community, country, and international levels (1). These measures include the lockdowns, curfews, and recently vaccination depending on the severity of the situation (1). The lockdown includes measures like home quarantine and avoiding any direct contact with any healthy or infected person, avoiding non-essential travel, observing social distancing rules like avoiding crowded public places and maintaining at least 2 m of distance between each person, closure of all learning institutions, cancellation of recreational venues, closing of public places and curfew (2, 3).

Lockdowns are short-term measures to reorganize, regroup, rebalance resources, and protect health workers who are in most cases overwhelmed by the high number of COVID-19 cases (4). However, prolonged impositions can be damaging. Lockdown is described as a hostile experience that can cause severe financial stress (5), social disorders such as social withdrawal, cyberbullying, alcohol misuse, and addiction; and mental health issues such as suicide attempts and depression (6). This is attributed to the separation from family and friends, loss of independence, lockdown length, monotonous lifestyle, lack of accurate information, and stigma (5). The enforcement of these stringent measures has significantly disrupted the lives of the student community by disorganization of their routines, leisure, examination postponement, graduation cancellations and increase in sedentary lifestyle. This exposes them to an increased risk of developing or deteriorating existing chronic health conditions such as major mental disorders.

This especially comes when the state of mental health among students is a developing area of concern. Various studies have indicated worrying data concerning the prevalence of depression and suicidal ideations among undergraduate students (7–10). Before the COVID-19 pandemic, the prevalence of depression among undergraduate students was reported at 21.5% (9). The restrictions set to curb the spread of COVID-19 including lockdowns not only deny people opportunities for social interaction, and pleasure but also put academic prospects of students in a turmoil which may collectively increase the risk of psychological distress and depression. Lockdowns also reduce the ability to seek help including mental health care services which may lead to detrimental outcomes such as severe depression, and suicidal ideations (8, 10). Therefore, this study aimed at assessing the prevalence of psychological distress and access to mental health services among undergraduate students in Uganda during the COVID-19 lockdown period.

## Methods

### Study Design and Setting

An online descriptive, cross-sectional study was undertaken during the second COVID-19 lockdown between August and September 2021. The study was conducted among undergraduate students enrolled in the following universities in Uganda: *P*ublic universities (Makerere University, Mbarara University of Science and Technology, Kyambogo University, Gulu University, and Busitema University) and private universities (Kampala International University (KIU), Uganda Christian University (UCU), Islamic University in Uganda (IUIU), St. Augustine (King Caesar) International University, and Ndejje University).

### Study Population

Eligible participants were undergraduate university students aged 18 years or older, able to access smartphones, and were within the social media channels namely WhatsApp Messenger and Facebook or Telegram were included in the study. The participants were excluded if they were not on any social media channels or could not access the internet. We could have performed a community-based national sampling survey, but it was not feasible due to the ongoing pandemic and lockdown measures to prevent the spread of COVID-19 by the time of data collection. Data was collected *via* an online Kobo toolbox®, which included an electronic informed consent form.

### Sample Size Calculation and Sampling

The sample size was calculated using Epi Info StatCalc for infinite population surveys. With a 5% acceptable margin of error, design effect of 1.0, cluster effect of 1.0, and a power of 80%, the estimated sample size at a 95% confidence interval (95% CI) was 384 participants. To cater for non-response associated with online surveys, 10% of the estimated sample size was added leading to a final sample size of 422 participants.

### Questionnaire Development

A well-researched and pre-validated questionnaire was used to collect information in three sections: sociodemographic characteristics (age, education details, tribe, ethnicity, sex, study program, year of study), the prevalence of psychological distress, and access to mental health services/ sources of mental health services.

### Measurements

#### Psychological Distress

It was assessed using a 44-word questionnaire adapted from the Distress Questionnaire-5 (DQ-5) which is a reliable and validated tool for assessing psychological distress among the general population. It is also useful in screening for common mental disorders in the general population. The participants indicated their level of agreement with the statements using responses, “Never” (1), “Rarely” (2), “Sometimes” (3), “Often” (4), or “Always” (5). A total score was then calculated by adding up each item score ranging from 5 to 25. Higher total scores were used to indicate greater psychological distress. Since this was a self-administered questionnaire, a cut-off of ≥14 was used to define psychological distress to increase specificity among study participants (11).

Questions adapted from the Patient Health Questionnaire-2 (PHQ-2) were also used to assess depression. The PHQ-2 is composed of two questions with four responses corresponding to scores from 0 to 3 consecutively. A PHQ-2 score ranges from 0 to 6 and the authors identified a score of 3 as the optimal cut point when using the tool to screen for depression.

The Cronbach's alpha for DQ-5 was 0.86 which is considered a good internal consistency while that PHQ-2 was 0.63.

#### Access to Mental Health Services

This was assessed by asking the participants to report their sources and means of access to mental health services for either mental health improvement, maintenance, or treatment.

#### Data Analysis and Management

Fully completed questionnaires were extracted from Kobo toolbox® and exported to a Microsoft Excel 2016 for cleaning and coding. The cleaned data was exported to STATA 16 for analysis. Numerical data were summarized as means and standard deviations or median and range as appropriate and categorical data was presented in figures and tables. Chi-square and Fischer's exact tests were used to assess the relationship between dependent and independent variables and multivariate logistic regression models were conducted after adjusting for confounders. A *p* < 0.05 was considered statistically significant.

## Results

### Characteristics of Participants

A total of 366 participants were enrolled in this survey. More than half (*n* = 232, 63.4%) of the participants were younger than 25 years with an overall mean age of 24.5 ± 4.6 years. Majority of the participants were male (*n* = 225, 61.5%), mostly from Makerere university (*n* = 119, 32.5%), and studying medical-related programs (*n* = 288, 78.7%). Fifty-four (14.8%) participants had ever tested for COVID-19, and 266 (72.7%) had a close friend or relative that had recently tested positive for COVID-19 (Table 1).

**Table 1:** Sociodemographic characteristics of participants.

**Variable *N* = 366**	**Frequency, *n* (%)**
**Age, mean** **±SD, years**	24.5 ± 4.6
<25 years	232 (63.4)
≥25 years	134 (36.6)
**Sex**	
Female	141 (38.5)
Male	225 (61.5)
**Religion**	
Christians	335 (91.5)
Moslem	20 (5.5)
Others	11 (3.0)
**Marital status**	
Married	36 (9.8)
Single	330 (90.2)
**Residence**	
Rural	85 (23.2)
Urban	281 (76.8)
**University**	
Busitema University	47 (12.8)
KIU	48 (13.1)
MUST	76 (20.8)
Makerere University	119 (32.5)
Others	76 (20.8)
Study program*	
Medical	288 (78.7)
Non-medical	78 (21.3)
**Personal history of COVID-19**	
No	312 (85.3)
Yes	54 (14.8)
**History of COVID-19 in a close friend/relative**	
No	100 (27.3)
Yes	266 (72.7)
**Access to mental health care services**	
No access	255 (61.5)
Have access	141 (38.5)

* *The study program defined a bachelor's degree a given participant was pursuing at the time of this study*.

### Psychological Distress Among Participants

The prevalence of psychological distress among the participants was 40.2% (*n* = 147). The mean DQ-5 score of 12.1 ± 4.6. Sex (*p* = 0.003) and study program (*p* = 0.046) were the factors significantly associated with psychological distress among the participants (Table 2). Female participants had 1.6-fold higher odds of having psychological distress compared to their male counterparts (aOR: 1.6, 95% CI: 1.0–2.6, *p* = 0.032).

**Table 2:** Bivariate analysis of factors associated with depression and psychological distress during COVID-19 lockdown.

**Variable**	**Psychological distress (DQ-5)**			**Depression (PHQ-2)**		
	**Psychologically distressed**	**Normal**	***p*-Value**	**Depressed**	**Normal**	***p*-Value**
**Overall**	**147 (40.2)**	**219 (59.8)**		**94 (25.7)**	**272 (74.3)**	
**Age**						
<25 years	100 (43.1)	132 (56.9)	0.131	67 (28.9)	165 (71.1)	0.066
≥25 years	47 (35.1)	87 (64.9)		27 (20.1)	107 (79.9)	
**Sex**						
Female	70 (49.6)	71 (50.4)	0.003	44 (31.2)	97 (68.8)	0.056
Male	77 (34.2)	148 (65.8)		50 (22.2)	175 (77.8)	
**Religion**						
Christians	138 (41.2)	197 (58.8)	0.304	88 (26.3)	247 (73.7)	0.079
Moslem	7 (35)	13 (65)		4 (20)	16 (80)	
Others	2 (18.2)	9 (81.8)		2 (18.2)	9 (81.8)	
**Marital status**						
Married	11 (30.6)	25 (69.4)	0.216	8 (22.2)	28 (77.8)	0.617
Single	136 (41.2)	194 (58.8)		86 (26.1)	244 (73.9)	
**Residence**						
Rural	31 (36.5)	54 (63.5)	0.428	24 (28.2)	61 (71.8)	0.539
Urban	116 (41.3)	165 (58.7)		70 (24.9)	211 (75.1)	
**University**						
Busitema University	21 (44.7)	26 (55.3)	0.703	15 (31.9)	32 (68.1)	0.180
KIU	21 (43.8)	27 (56.3)		14 (29.2)	34 (70.8)	
MUST	33 (43.4)	43 (56.6)		23 (30.3)	53 (69.7)	
Makerere University	42 (35.3)	77 (64.7)		21 (17.6)	98 (82.4)	
Others	30 (39.5)	46 (60.5)		21 (27.6)	55 (72.4)	
**Study program**						
Medical	108 (37.5)	180 (62.5)	0.046	63 (21.9)	225 (78.1)	0.001
Non-medical	39 (50)	39 (50)		31 (39.7)	47 (60.3)	
**Personal history of COVID-19**						
No	127 (40.7)	185 (59.3)	0.612	81 (26)	231 (74)	0.769
Yes	20 (37)	34 (63)		13 (24.1)	41 (75.9)	
**History of COVID-19 in a close friend/relative**						
No	32 (32)	68 (68)	0.051	27 (27)	73 (73)	0.724
Yes	115 (43.2)	151 (56.8)		67 (25.2)	199 (74.8)	
**Access to mental health care services**						
No access	106 (47.1)	119 (52.9)	0.001	70 (31.1)	155 (68.9)	0.003
Have access	41 (29.1)	100 (70.9)		24 (17)	117 (83)	

### Depression Among Participants

The prevalence of depression was 25.7% (*n* = 94). The mean PHQ-2 score was 1.7 ± 1.6.

The study program was found to be one of the factors significantly influencing depression among undergraduate students (Table 2). Participants pursuing non-medical programs at their respective universities had two-fold higher odds of being depressed compared to those doing a medical program (aOR: 2.2, 95% CI: 1.3–3.7, *p* = 0.005), Table 3.

**Table 3:** Multivariate logistic regression of the factors associated with psychological distress and depression during COVID-19 lockdown.

**Variable**	**Psychological distress (DQ-5)**		**Depression (PHQ-2)**	
	**aOR (95% CI)**	***p*-Value**	**aOR (95% CI)**	***p*-Value**
**Age**				
<25 years	1			
≥25 years	0.9 (0.6–1.5)	0.704	0.8 (0.45–1.4)	0.393
**Sex**				
Male	1.0			
Female	1.6 (1–2.5)	0.044	1.4 (0.8–2.2)	0.238
**Religion**				
Christian	1.0			
Moslem	0.9 (0.3–2.4)	0.813	0.8 (0.2–2.4)	0.630
Others	0.3 (0.1–1.6)	0.16	0.7 (0.1–3.6)	0.707
**Study program**				
Medical	1.0			
Non-medical	1.3 (0.8–2.3)	0.272	2.0 (1.1–3.4)	0.016
**History of COVID-19 in a close friend/relative**				
No	1.0			
Yes	1.6 (1–2.7)	0.066	0.9 (0.5–1.5)	0.671
**Access to mental health care services**				
No access	1		1	
Have access	0.5 (0.3–0.8)	0.005	0.6 (0.3–0.9)	0.034

### Access to Mental Health Care Services During COVID-19 Lockdown

**Figure 1:**
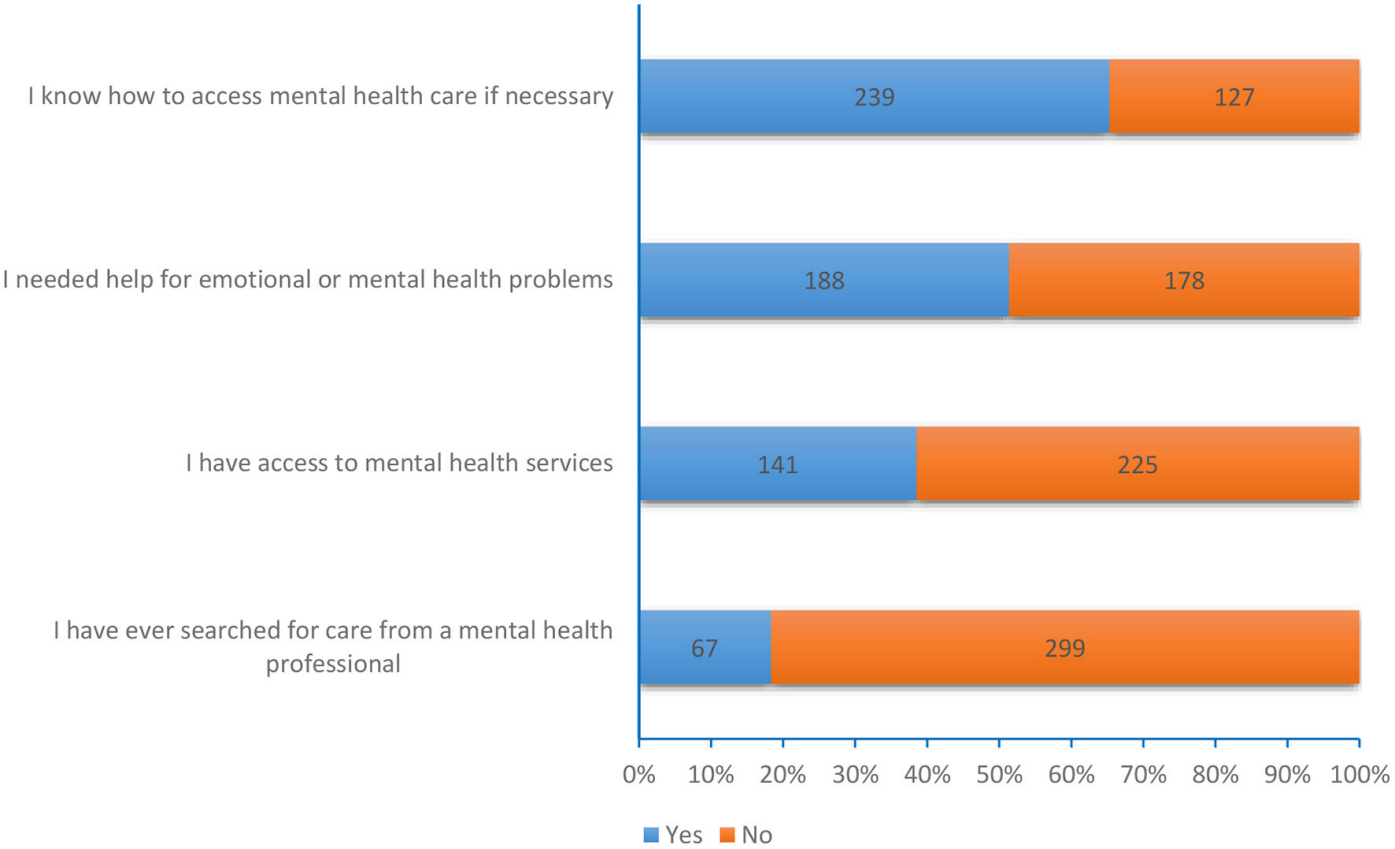
Access to mental health services in the last 12 months of COVID-19 lockdown in Uganda.

The majority (*n* = 239, 65.3%) of the participants reported knowing how to access mental health care and almost half (*n* = 188, 51.4%) reported having needed emotional support. Only 67 (18.3%) participants had ever sought care from a mental health professional (Figure 1). Participants who had access to mental health care were less likely to be psychologically distressed (aOR: 0.5, 95%CI: 0.3–0.8, *p* = 0.005) or depressed (aOR: 0.6, 95%CI: 0.3–0.9, *p* = 0.034; Table 3).

**Figure 2:**
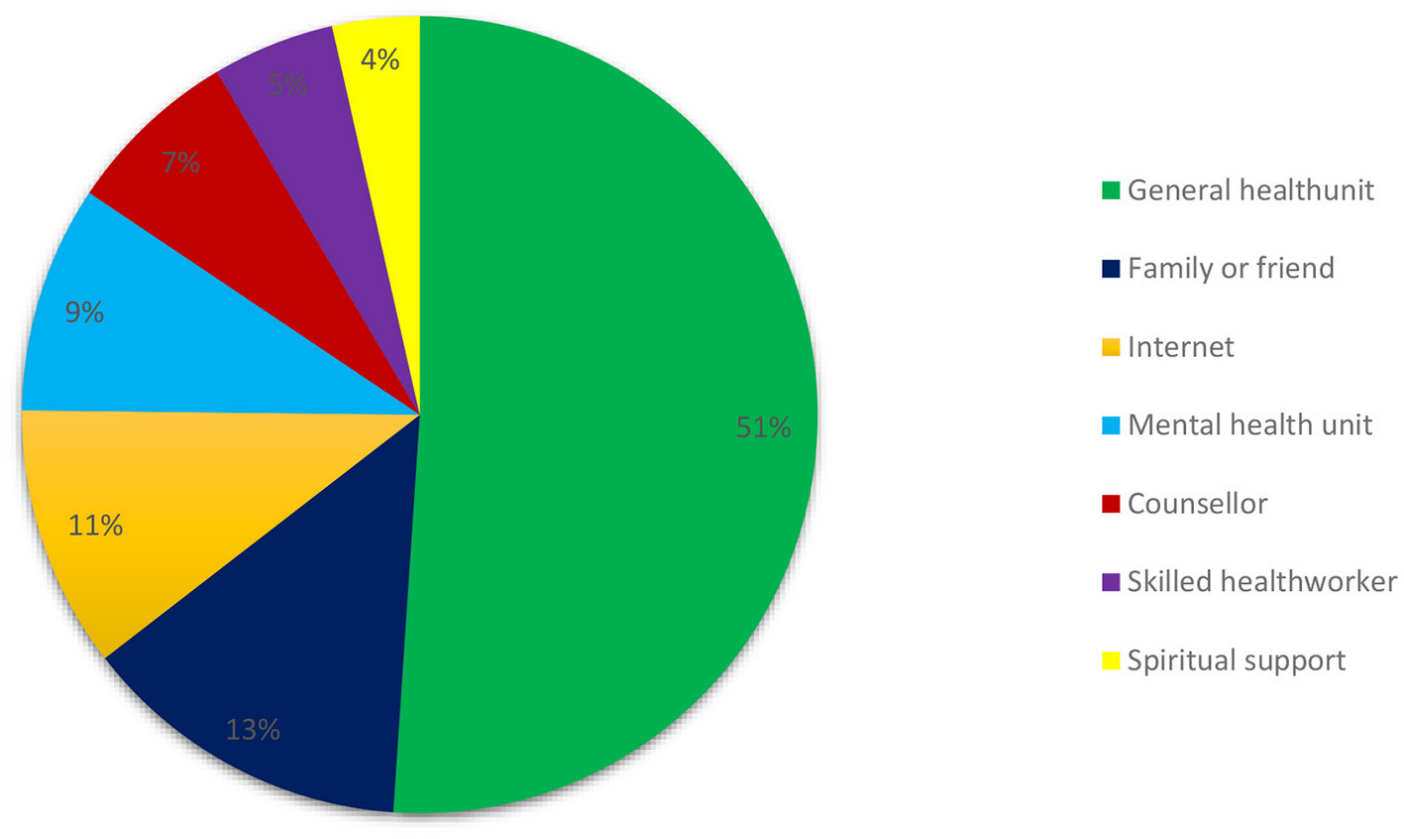
Reference points and contact persons in case of need to access mental health care as outlined by participants.

Of the 141 participants who had access to mental healthcare services during the lockdown, only 10 (7%) accessed a counselor and 13 (9%) accessed a mental health unit (Figure 2).

**Figure 3:**
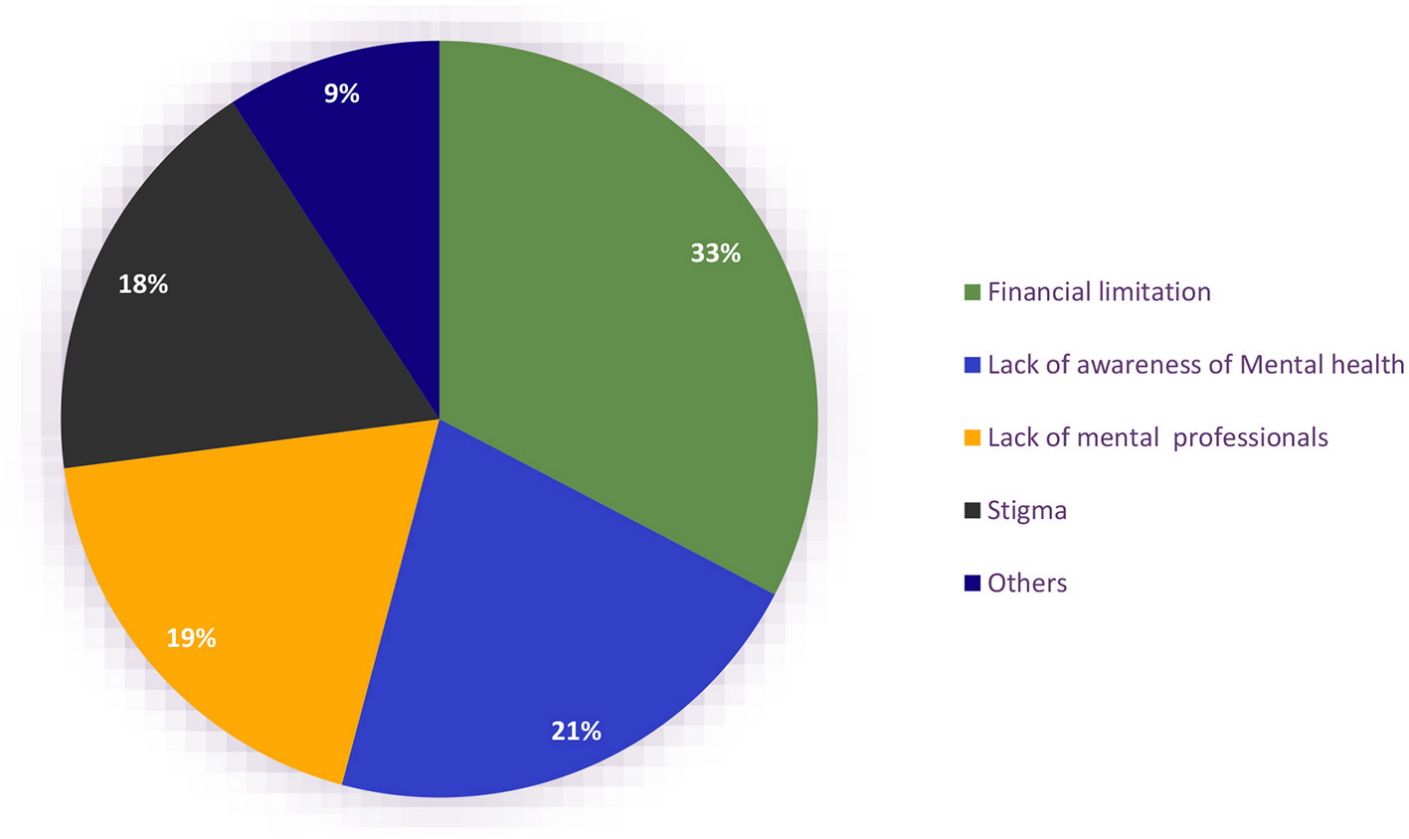
Barriers to the accessibility of mental health services in Uganda during the COVID-19 lockdown.

The most common barriers to accessibility to mental health care included financial limitations (*n* = 181, 49.5%), lack of awareness of mental health (*n* = 119, 32.5%), lack of mental health professionals (*n* = 104, 28.4%), and stigma (*n* = 51, 13.9%; Figure 3).

## Discussion

In this study, we investigated the prevalence of psychological distress, depression, and access to mental health care services among undergraduate students during the COVID-19 lockdown in Uganda. Our findings suggest that almost half of the participants experienced psychological distress during the lockdown irrespective of their COVID-19 test results. Secondly, one-fourth of the participants experienced depression during the lockdown. These findings confirm the negative impact of lockdown on the mental health of undergraduate university students in Uganda.

Results from our study were only slightly higher than those reported in previous studies about depression in Uganda. However, psychological distress is rarely studied and to the best of our knowledge, this is the first study describing it among Ugandan undergraduates. A cross-sectional study to establish the prevalence and factors associated with depression among medical students at Makerere University College of Health Sciences, Uganda reported a 21.5% prevalence of depression (7). Likewise, the prevalence of depression was found to be 21% among adolescents in central Uganda but before lockdown (12). The prevalence of depression in our study though slightly higher compared to the previous studies in Uganda was not surprising given that it was conducted during the lockdown; a period considered stressful both mentally and emotionally as documented in other studies.

Internationally our results aligned with those reported in a cross-sectional web-based survey among university students in United Arab Emirates where 51% were in psychological distress (13) during the COVID- 19 pandemic as well as reported in the United Kingdom (14). On the contrary, a study in Italy among University students during the COVID-19 lockdown found only 21.4% to have experienced lockdown as a traumatic experience with 26% experiencing depressive symptoms (15).

With interest in Sub-Saharan Africa, our results are congruent with those reported from a cross-sectional study among a group of university students in Ethiopia where 53.2% of the participants were found to have mental distress, and the female students were more likely to be mentally distressed compared to male students. This was explained in terms of the susceptibility to stressors due to domestic violence and hormonal changes during menstruation as well as the structural determinants of mental health such as income and social rank/roles of women (16).

However, the difference in geographical locations and political—social living conditions as well as modes of investigation of psychological distress could explain the varying results. The different varying tools employed include the Self-Reporting Questionnaire-20 (SRQ-20) (16), Mini International Neuro-Psychiatric Interview for Children and Adolescents 2.0 (MINI-KID) (12), and Patient Health Questionnaire 9 (PHQ9) (7) whereas we used the DQ-5 and PHQ-2. This hence could account for the differences in results due to differences in sensitivity and specificity of the tools.

Besides gender, the study program was also a factor significantly influencing psychological distress among undergraduate students where participants who were doing a non-medical program at their respective universities, were twice as more likely to be depressed as those doing a medical program. There is a low-evidence working theory both nationally (17) and internationally (18) that medical students are prone to more psychological distress than their counterparts. The observed difference in this case, maybe explained by the timely opening of medical schools in Uganda during lockdown as opposed to nonmedical colleges hence giving medical students hope for continued studies and a means of productive engagement possibly reducing the risk of psychological distress as compared to their counterparts. Additionally, medical students are presumed to be oriented about early recognition of signs of psychological distress, risk factors, and measures to avoid them. In a similar study in UAE, psychological distress was found to be more associated with students with a history of mental illness, older students, students who exhibited anxiety concerning COVID-19 anxiety and fear, and those who spent more than 4 h reading about COVID-19 (13). An Italian study found the increase in length of home confinement to increase the likelihood of experiencing posttraumatic symptomatology by over three times (15).

Despite such demand, access to mental health has not been satisfactorily discussed in Uganda. A recent study described Uganda's mental health system as one with services disproportionately concentrated around the capital, with those living in the country having little access to mental healthcare. They described barriers to access to mental health as low funding as per international standards, high demand for services vs. supply, stigma, and traditional beliefs regarding causation that inhibit seeking mental health services. Among others were the high poverty rates rendering people too poor to pay for travel to clinics or the costs of medication as well as the poor living conditions at the national mental health facility (19).

Similarly, out of the 65% of the participants who reported knowing how to access mental health services and 51.4% who reported having needed emotional support, only 18.3% had ever sought care from a mental health professional. That said, among those who reported having access, only 7 and 9% were having access to a counselor and a mental health unit respectively. In line with the previous report, almost half of the participants cited financial limitations as a barrier to their access to mental health services in addition to stigma and lack of mental health awareness and professionals. Moreover, it was found in this study that having access to mental health care services may offer significant protection against psychological distress and depression. The role of accessibility to mental health services in protection against these challenges may be visualized in two ways; (1) it indirectly confirms that mental health is rather a disease than otherwise postulated which may reduce stigma among the general population, (2) early identification of mental illnesses is possible with prompt management. Therefore, more needs to be done in the development of the mental health sector in Uganda including financial input to support infrastructure development and revision of policies, especially in emergency times like this COVID-19 pandemic.

Although only 14.8% of the participants had ever tested for COVID-19, the majority, (72.7%) had a close friend or relative that had tested positive in the recent past indicating the high COVID-19 disease experience. These findings are indicative of the raised prevalence of psychological distress and the several barriers to access to mental health services among university students, especially during this lockdown. These findings also highlight the gender-specific psychological distress prevalence hence bringing forth a need for investigation of gender-specific risk factors and interventions to address them.

### Limitations

Several limitations should be taken into consideration as the reader interpret these findings. First, the cross-sectional design employed in our study does not allow for a causal interpretation of the results. Secondly, since the study was conducted at the time when the country was battling the second wave of COVID-19 and in total lockdown, data collection was only possible *via* online methods hence missing out on the population that did not own smartphones, those with poor connectivity to the internet or could not meet the data costs for participating in this study. This could lead to selection bias threatens the ability to generalize study findings. Third, since the questionnaire was self-administered, there was a possibility of obtaining correct answers without fully understanding the questions, recall bias and participants may have interpreted the questions differently. However, being an anonymous self-administered questionnaire could have allowed participants to answer sensitive questions with honesty without fear of being judged.

## Conclusion

Among university students in Uganda during the COVID-19 lockdown, the burden of psychological distress and depression was substantial. However, access to mental health services was limited by several factors such as financial limitations, stigma, cultural beliefs, and a limited number of mental health care professionals.

## Data Availability Statement

The original contributions presented in the study are included in the article/supplementary files, further inquiries can be directed to the corresponding author/s.

## Ethics Statement

The studies involving human participants were reviewed and approved by Mulago Hospital Research and Ethics Committee. The participants provided their written informed consent to participate in this study.

## Author Contributions

All authors made substantial contributions to conception and design, acquisition of data, or analysis and interpretation of data, took part in drafting the article or revising it critically for important intellectual content, agreed to submit it to the current journal, gave final approval to the version to be published, and agree to be accountable for all aspects of the work.

## Conflict of Interest

The authors declare that the research was conducted in the absence of any commercial or financial relationships that could be construed as a potential conflict of interest.

## Publisher's Note

All claims expressed in this article are solely those of the authors and do not necessarily represent those of their affiliated organizations, or those of the publisher, the editors and the reviewers. Any product that may be evaluated in this article, or claim that may be made by its manufacturer, is not guaranteed or endorsed by the publisher.
